# Evaluation of an automated haemolytic method for the determination of anti-streptolysin O antibodies

**DOI:** 10.1155/S1463924685000220

**Published:** 1985

**Authors:** F. Parri, E. de Majo

**Affiliations:** Laboratorio di Batteriologia e Virologia Ospedale Careggi U.S.L. 10D Via della Quiete 8 Firenze Italy


Journal of Automatic Chemistry/*Journal of Clinical Laboratory Automation*, Vol. 7, No. 2 (Apr–Jun 1985), pp. 99–101

### Evaluation of an automated haemolytic method for the determination of anti-streptolysin O antibodies

**F. Parri and E. de Majo**

*Laboratorio di Batteriologia e Virologia, Ospedale Careggi U.S.L. 10D, Via della Quiete 8, Firenze, Italy*

### 1. Introduction

Streptolysin O (SO) is an exotoxin produced by the majority of group A, C, and G beta-haemolytic streptococci, Groups C and G, however, rarely cause diseases in man, so finding high anti-Streptolysın O (ASO) titer is an index of group A beta-haemolytic streptococcal infection. This is of great clinical help because serious complications may follow this kind of infection.

ASO titer determination is a currently used laboratory procedure for the diagnosis of streptococcal infections; many authorities agree that high ASO titers will be found in about 80% of streptococcal infections.

The ASO titer is generally carried out by the haemolytic method of Rantz and Randall [1] or modifications of this method, which requires a series of steps (sample inactivation, preparation of a series of sample dilutions, preparation of a 5% rabbit erythrocyte suspension) which limit its usefulness. Further, it has a critical point due to the poor stability of reduced SO in the presence of oxygen with consequent loss of lytic activity.

Recently, more useful methods have been devised. Some kits on the market make use of the agglutination of latex particles or formol-treated erythrocytes as support for SO. Other kits (Aso-Quantum, Sclavo, I 53100 Siena, Italy, and Taso-tec/Taso-matic Diesse, Diesse, I 53035 Monteriggioni, Italy) are based on the haemolytic method and use oxidized SO, which is without lytic activity and hence capable of binding the specific antibodies. The antigen-antibody reaction is detected after the addition of a reducing agent, which makes the unbound SO once again capable of lysing the erythrocytes [2, 3 and 4].

Aso-Quantum is a manual method which may be used with whole blood since it employs the patient's own erythrocytes as detectors of the antigen-antibody reaction. This means, however, that the determination must be performed within 24 h of the blood sample being taken. Since laboratories are not always in a position to do this, modifications of the original method have been developed which allow the use of the patient's serum together with human (O group and Rh negative) or rabbit erythrocytes [5 and 6].

Taso-tec/Taso-matic is an automated method [7], which can be used both on undiluted whole blood or serum; in the latter case, fresh human erythrocytes are used as detectors by adding them directly to oxidized SO. The rate of haemolysis is followed photometrically on a Taso-matic instrument in which a control containing only SO, erythrocytes and reducing reagent allows more accurate results and greater standardization to be obtained. In the presence of known levels of antibodies a series of sigmoid curves are obtained with slopes inversely proportional to the concentration of antibodies (see figure 1). It is therefore possible to establish a correlation between the time required to reach a 10% reduction of the initial absorbance and the level of antibodies in the sample [7]. Taso-matic equipment takes a first reading at $t_0$ (the time after the reducing agent is automatically added) and then measures until $t_x$ is reached (the time at which the absorbance becomes 90% of the initial value). According to the time between $t_0$ and $t_x$, the instrument calculates the ASO titer of each sample and prints them directly in International Units (IU). The possibility of introducing a control serum at each analysis cycle allows a true quality-control programme to be carried out. The Taso-matic can take up to 17 serum sample cuvettes and performs automatic stirring, thermoregulation, addition of the reducing agent to each cuvette, data storage,

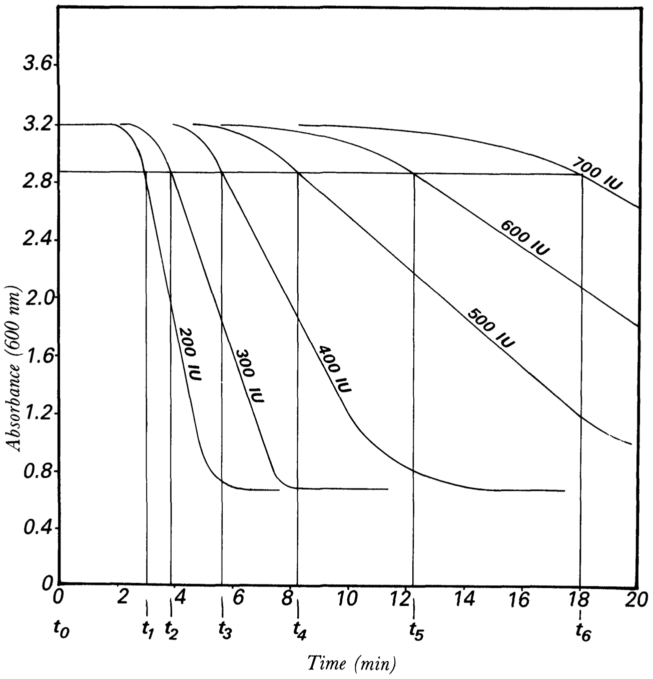

*Figure 1. Haemolysis curves for samples as known ASO titer. Where $t_0$ = time when the reducing reagent was added and $t_1$, $t_2$, $t_3$, $t_4$, $t_5$, $t_6$ = time at which a 10% decrease in the initial absorbance value due to haemolysis was obtained, for samples having 200, 300, 400, 500, 600 and 700 IU of ASO titer.*

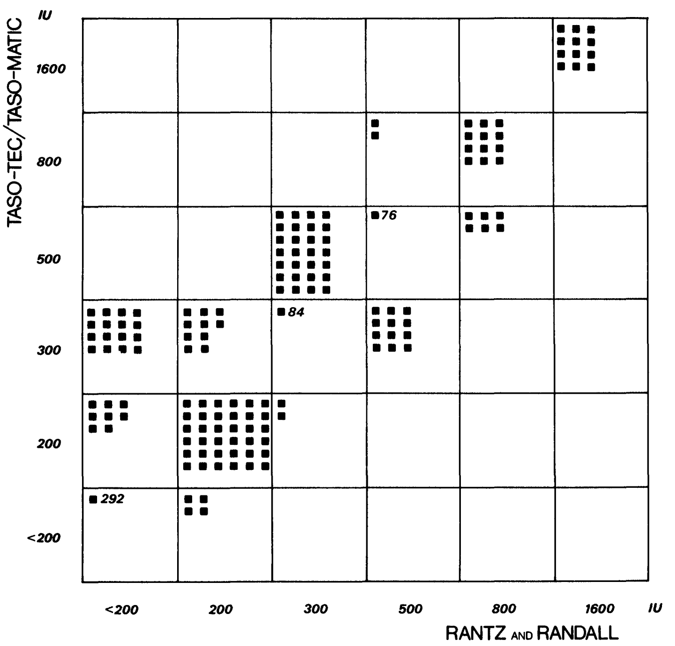

*Table 1. Correlation between the Rantz and Randall method and Taso-tec/Taso-matic automated method.*

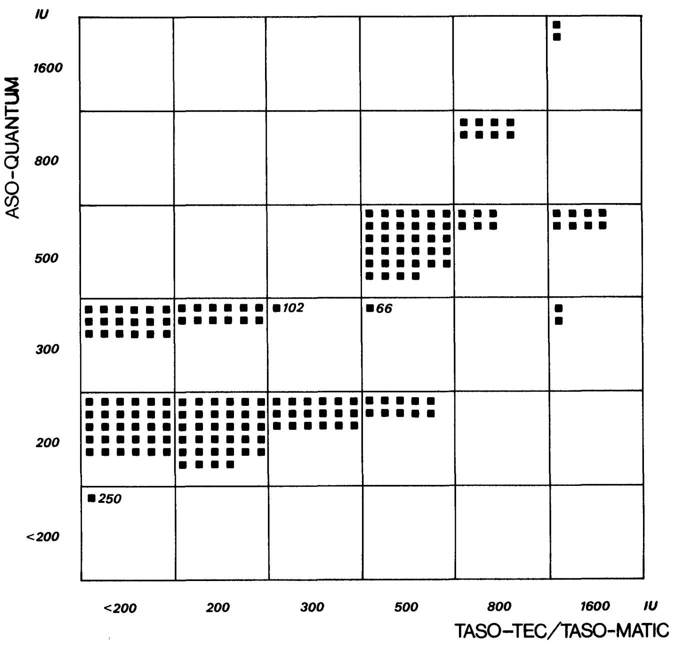

*Table 3. Correlation between Taso-tec/Taso-matic and Aso-Quantum methods.*

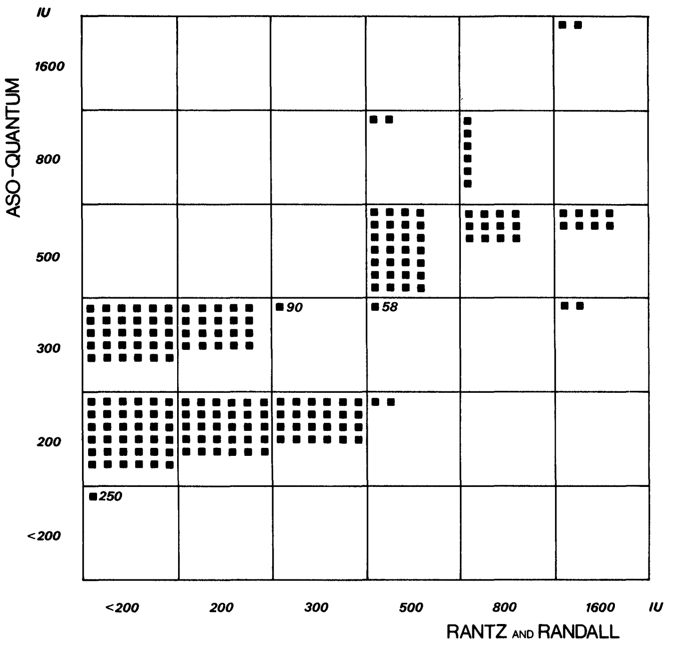

*Table 2. Correlation between the Rantz and Randall and Aso-Quantum methods.*

processing, and printing-out of results in IUs within 30 min. The Rantz and Randall method requires a serial dilution of each sample, while Aso-Quantum needs serial dilution of SO. Taso-tec/Taso-matic method performs each determination in one cuvette with a single dose of SO.

### Materials and methods

The evaluation was done on 600 serum samples sent to the laboratory for ASO titer determination. The Rantz and Randall method was used as a reference and the Taso-tec/Taso-matic method using human erythrocytes, was compared to the reference and to the Aso-Quantum methods utilizing rabbit erythrocytes. All the methods were performed according to the manufacturers' technical instructions.

### Results

Table 1 compares the results obtained with the Rantz and Randall and Taso-tec/Taso-matic methods. Statistical analysis of the results shows an excellent agreement between the two procedures ($y = 22{\cdot}23 + 0{\cdot}95x$; $r = 0{\cdot}96$), indeed the correlation coefficient $r = 0{\cdot}96$ does not differ significantly from 1 ($p<0{\cdot}05$).

Table 2 shows the correlation between the Rantz and Randall and the Aso-Quantum methods. Statistical analysis of the results indicates less agreement between methods ($y = 127{\cdot}15 + 0{\cdot}42x$; $r = 0{\cdot}73$); the value of the intercept (127·15) and the value of the slope (0·42) indicate that the Aso-Quantum gives lower results than the reference method at high levels, and higher results at low levels.

Table 3 compares the results obtained with the Taso-tec/Taso-matic and Aso-Quantum methods. Statistical analysis of the results shows a poor correlation between the methods ($y = 121{\cdot}76 + 0{\cdot}43x$; $r = 0{\cdot}73$) and indirectly confirms the good correlation between the reference and the Taso-tec/Taso-matic methods.

### Discussion

The well correlated results with the reference method and the easy-to-use equipment, mean that the Taso-tec/Tasomatic system is useful for clinical laboratories as a valid alternative to the traditional, manual method for determining ASO titer. Important assets are its ease of use and speed and the possibility of performing a complete quality control program.

### References

1. Rantz, L. A. and Randall, E., *Proceedings of the Society of Experimental Medicine,* **59** (1945), 22.
2. Alouf, J. E. and Raynaud, E. M., *Annales de l'Institut Pasteur,* **114** (1968), 812.
3. Bernheimer, A. W., *Biochemica & Biophysica Acta,* **344** (1974), 27.
4. Bernheimer, A. W. and Avigad, L. S., *Infection and Immunity,* **1** (1970), 509.
5. Ricci, A., Berti, B., Moauro, C., Porro, M., Neri, P., and Tarli, P., *Journal of Clinical Microbiology,* **8** (1978), 263.
6. Cualbu Diez, G., *Quaderni Sclavo di Diagnostica,* **16** (1980), 312.
7. Ricci, A. *Hemolytic method for the kinetic determination of antistreptolysin O antibodies in blood or serum samples, using oxidized streptolysin O* (U.S. Patent No. 4, 379,850).

**35th CANADIAN CHEMICAL ENGINEERING CONFERENCE**

*Calgary, Alberta, 6–9 October 1985*

Organized by the Canadian Society for Chemical Engineering/Société Canadienne du Génie Chimique, the conference will be held at the Calgary Convention Centre, and will consist of seven concurrent technical and general-interest sessions. The papers will cover a wide range of topics from fundamentals to industrial applications of chemical engineering. There will also be sessions relevant to the chemical, process, and energy industries. Several sessions, including one on government relations, will include invited speakers. The economic and Business Management Division (EBM) of the Chemical Institute of Canada is co-sponsoring and organizing several sessions on forecasts, forecasting and planning, petrochemicals, and the business side of large projects.

Technical sessions at the conference are planned on the following subjects:

Biotechnology
Business side of large projects (EBM)
Chemical engineering fundamentals with applications
Chemical processing
Coal, oil and tar sands
Cogeneration
Computer aided design
Computer control
Entrepreneurs in chemical engineering
Environmental opportunities
Environmental regulations
Forecasts, forecasting and planning (EBM)
Government relations
Petrochemical outlook (EBM)
Plastics and materials
The gas plant industry
Use of PCs in chemical engineering
Utilization of methane.

*Further information from Roger M. Butler, Department of Chemical and Petroleum Engineering, University of Calgary, Calgary, Alberta T2N IN4, Canada. Tel.: 403 284 7133.*

